# Papillary Thyroid Carcinoma in the Era of De-Escalation: Toward Personalized and Less Aggressive Management

**DOI:** 10.3390/cancers18081317

**Published:** 2026-04-21

**Authors:** Joaquin Gomez-Ramirez, Raquel Arranz Jiménez, Beatriz López de la Torre, Elisa York Pineda, Paola Parra Ramírez

**Affiliations:** 1 Endocrine Surgery Unit, Department of General and Digestive Surgery, Instituto de Investigación Hospital Universitario La Paz (IdiPAZ), 28029 Madrid, Spain; 2 Endocrinology and Nutrition Department, Hospital Universitario La Paz, 28046 Madrid, Spain

**Keywords:** thyroid cancer, papillary thyroid cancer, active surveillance

## Abstract

**Background:** Papillary thyroid carcinoma (PTC) is the most common type of thyroid cancer and is generally associated with an excellent prognosis. Historically, treatment strategies were uniform and frequently aggressive, including total thyroidectomy and routine radioiodine ablation, even in low-risk cases. **Current Perspective:** Over the past decade, the management of PTC has shifted toward a de-escalation paradigm. This transition is driven by high evidence showing that the majority of PTCs follow an indolent course, with low recurrence and mortality rates. As a result, there is increasing emphasis on tailoring the extent of surgery and adjuvant therapy to individual patient risk profiles. Active surveillance, hemithyroidectomy, and selective use of radioiodine now represent valid alternatives to traditional radical approaches, particularly for low-risk tumors. **Clinical Implications:** The goal of this evolution is to balance oncologic safety with quality of life, reducing overtreatment and minimizing long-term complications such as hypoparathyroidism or recurrent laryngeal nerve injury. Personalized treatment decisions are now guided by tumor biology, molecular markers, and refined risk stratification systems. **Conclusions**: This article will review the current evidence supporting this shift, highlight the challenges of implementation in clinical practice, and discuss future trends in the management of papillary thyroid carcinoma.

## Simple Summary

Papillary thyroid carcinoma (PTC), the most prevalent form of thyroid malignancy, is typically associated with an excellent prognosis. Historically, management involved uniformly aggressive strategies, including total thyroidectomy and routine radioiodine ablation, even in low-risk cases. Over the past decade, a paradigm shift towards de-escalation has emerged, supported by robust evidence demonstrating the indolent nature of most PTCs, with low rates of recurrence and mortality. Contemporary management emphasises risk-adapted, individualised care, incorporating options such as active surveillance, hemithyroidectomy, and selective radioiodine use, particularly for low-risk disease. This approach aims to maintain oncological safety while preserving quality of life and reducing treatment-related morbidity, including hypoparathyroidism and recurrent laryngeal nerve injury. Decision-making is increasingly informed by tumour biology, molecular profiling, and refined risk stratification systems, with ongoing challenges in clinical implementation and future optimisation.

## 1. Introduction

Papillary thyroid carcinoma (PTC) accounts for approximately 85% of all thyroid cancers arising from thyroid follicular cells and has shown a steady increase in incidence over recent decades. PTC currently has an excellent prognosis, with a mortality rate of only 2–4% beyond 20 years. However, the recurrence rate remains relatively high, ranging from 10% to 30% after more than 10 years of follow-up. At present, most newly diagnosed PTCs fall into the low-risk category, corresponding to small tumors with low biological aggressiveness. Distant metastases are uncommon, occurring in a small proportion of patients, and when present, they most frequently involve the lungs and, less commonly, the skeletal system. Bone metastases from PTC are rare, and involvement of the maxillofacial region, particularly the mandible, is exceptional and only sporadically reported in the literature. These atypical metastatic presentations may pose significant diagnostic challenges due to their nonspecific clinical and radiological features. Furthermore, the management of advanced or metastatic PTC can be complex, requiring a multidisciplinary approach, especially in cases with unusual metastatic sites or limited response to conventional therapies.

During the 20th century, PTC was treated under a uniform and aggressive paradigm, partly influenced by the landmark work of Mazzaferri [1]. Standard practice consisted of total thyroidectomy followed by radioactive iodine (RAI) ablation and long-term thyroid-stimulating hormone (TSH) suppression [2]. This approach achieved low recurrence rates and high survival but was associated with significant risks, including surgical morbidity, lifelong hormone dependence, and radiation-related adverse effects. The advent of advanced imaging technologies and the increased incidental detection of microcarcinomas (<1 cm) revealed the biological heterogeneity of PTC, challenging the need for universally aggressive treatment strategies [3,4,5].

The 2015 American Thyroid Association (ATA) guidelines [6] marked a turning point in clinical practice. For the first time, thyroid lobectomy was validated as an appropriate option for tumors ≤ 4 cm confined to the thyroid gland, without extrathyroidal extension or clinically evident lymph node metastases. A selective approach to RAI use was recommended, reserving it for patients with high-risk disease or aggressive histological features. In addition, dynamic risk stratification was introduced as a tool to personalize surveillance and follow-up by evaluating response to initial therapy. This model adjusts the intensity of follow-up and treatment according to the patient’s initial response: patients with an excellent response may undergo less intensive monitoring, whereas those with an incomplete response require closer surveillance or additional intervention. This individualized approach optimizes resource use and minimizes unnecessary interventions. This has been reinforced in the latest update of these guidelines (2025), as will be discussed later, where lobectomy is once again highlighted as the treatment of choice for low-risk tumors and a selective use of radioiodine therapy is emphasized [7].

As mentioned above, an important change introduced by these guidelines concerns RAI therapy, which was previously considered standard but is now increasingly viewed as a selective treatment. In low-risk patients, RAI does not appear to provide a significant benefit in terms of recurrence or survival, as demonstrated by the ESTIMABL2 trial [8] and its recent 5-year update [9]. Consequently, RAI should be reserved for tumors with extrathyroidal extension, positive lymph nodes, or high-risk histological features. This shift reduces radiation exposure, long-term adverse effects, and healthcare costs [10].

## 2. Epidemiology and Genetics

The main risk factors for PTC include exposure to ionizing radiation during childhood, genetic predisposition, and, to a lesser extent, dietary iodine variations. In addition, obesity is increasingly recognized as a risk factor, with epidemiological evidence showing a positive association between higher body mass index and the risk of thyroid cancer, particularly PTC [11].

Over recent decades, several genetic polymorphisms and molecular alterations—such as BRAF V600E, RET/PTC rearrangements, RAS mutations, and TERT promoter mutations—have been identified, with important prognostic implications that are now incorporated into personalized patient assessment.

In 2014, The Cancer Genome Atlas (TCGA) published a pan-genomic analysis of the largest PTC cohort to date (n = 496), excluding poorly differentiated and undifferentiated carcinomas. TCGA classified PTC into two major molecular subtypes: BRAFV600E-like (BVL) and RAS-like (RL), introducing a molecular classification that complements traditional histopathological classification. This analysis identified novel genetic alterations in known oncogenic drivers, as well as new drivers such as EIF1AX, PPM1D, and CHEK2, reducing the proportion of PTCs of unknown origin from 25% to 3.5% [12].

In general, PTC is characterized by alterations in the MAPK (mitogen-activated protein kinase) signaling pathway, which promotes cellular proliferation. The BRAF V600E mutation, present in 40–60% of cases, leads to constitutive activation of this pathway, although its prognostic impact depends on the presence of co-mutations. The PI3K/AKT pathway may also be altered, particularly in more aggressive or poorly differentiated subtypes, reflecting a molecular transition toward radioiodine-refractory phenotypes. Recent studies demonstrate that the coexistence of BRAF V600E and TERT promoter mutations is associated with an increased risk of recurrence and mortality. Integration of these findings into prognostic algorithms has fostered the concept of integrated molecular risk, in which genotype modulates therapeutic intensity [13,14].

## 3. Staging and Prognosis

Currently, the primary goal of PTC staging is not so much to predict mortality risk—which is fortunately low in most cases—but rather to enable a more individualized, tailored treatment approach, adjusting follow-up according to disease evolution and therapeutic response. Historically, various staging systems based on prognostic risk factors have been used in PTC, but early classifications did not consider other relevant clinicopathological features, such as lymph node involvement, histological subtype, disease course, extent of surgery, or treatment response, often resulting in overtreatment.

Although the AJCC/UICC TNM system and the MACIS score have been widely used, most patients with PTC have excellent overall survival, making the prediction of persistent or recurrent disease the main clinical concern. Accordingly, the ATA guidelines in 2009 and 2015 established a risk stratification system—largely consistent with other scientific societies—dividing patients into low-, intermediate-, and high-risk categories.

Recurrence risk is based on tumor characteristics such as size, histological subtype, presence of lymph node metastases, extrathyroidal extension into perithyroidal soft tissues, size of lymph node metastases (≤2 mm or ≥3 cm), and number of involved lymph nodes (<5 or >5) (Table 1). As discussed later, this recurrence risk categorization has important implications for the indication of adjuvant RAI therapy.

Dynamic risk assessment is also essential and should incorporate treatment-related variables. Response to therapy, evaluated through thyroglobulin (Tg), anti-thyroglobulin antibodies (TgAb), and imaging studies, should be continuously reassessed according to patient risk, providing real-time prognostic information.

Regarding histological classification, the World Health Organization (WHO) published a new edition in 2022 [15]. A major change from previous editions is the exclusion of invasive follicular variant PTC from the classic variants, recognizing it as a distinct entity due to its intrinsic features that place it in an intermediate category between PTC and follicular thyroid carcinoma. In addition, noninvasive follicular thyroid neoplasm with papillary-like nuclear features (NIFTP), already included in the 2017 classification and considered a tumor of very low malignant potential with indolent behavior, further confirms its low aggressiveness when correctly diagnosed. Furthermore, papillary thyroid carcinoma is now recommended to be called micropapillary tumor [15].

Within PTC, in addition to the classic variant, several clinically relevant subtypes exist. Among these, the tall cell (TC), columnar cell (CC), and hobnail (HN) variants exhibit more aggressive behavior compared with classic PTC and are therefore classified as at least intermediate-risk tumors in most risk stratification systems. Other subtypes include the diffuse sclerosing variant, which has a higher tendency toward multifocality, extrathyroidal extension, and lymphatic spread, and the solid/trabecular subtype, which may occasionally behave similarly to poorly differentiated carcinoma.

## 4. Therapeutics Options

### 4.1. Active Surveillance

Advances in diagnostic techniques—particularly ultrasound—have resulted in a high proportion of patients being diagnosed with PTC tumors < 10 mm (papillary microcarcinomas or, more recently, micropapillary tumors). Active surveillance, initially implemented in Japan [16,17], has become established as a safe strategy for low-risk papillary microcarcinomas, especially in elderly patients with solitary tumors, well-characterized on ultrasound, and with access to structured and active follow-up.

Management algorithms are based on clinical and histological characteristics:

- **Ideal candidates for active surveillance:** elderly patients with solitary, intrathyroidal tumors and well-defined margins.
- **Potential candidates for active surveillance:** middle-aged patients, multifocal tumors, lesions adjacent to the thyroid capsule, not close to a recurrent laryngeal nerve, and no histology associated with poorer prognosis.
- **Patients in whom observation is inappropriate:** subcapsular tumors in critical locations (near the recurrent laryngeal nerve or trachea), extrathyroidal extension, lymphadenopathy, distant metastases, or evidence of tumor growth during follow-up.

Studies with follow-up exceeding 10 years have shown that most tumors remain stable, with a low risk of progression. This approach requires clear communication regarding risks and benefits, rigorous ultrasound surveillance, and patient commitment, and has been shown to preserve quality of life by avoiding unnecessary surgery. Active surveillance has been adopted by multiple scientific societies and is included as an option in current guidelines for selected patients [18]. Importantly, this strategy does not preclude surgical treatment but rather postpones it until tumor growth or complications occur, which happens in only 3–5% of patients over 70–80 years of age diagnosed with papillary microcarcinoma [19].

### 4.2. Extent of Surgical Treatment

Total thyroidectomy was the standard treatment for decades for three historical reasons: facilitating RAI ablation, optimizing thyroglobulin-based follow-up, and eliminating contralateral risk. However, multiple studies over the past decade have demonstrated that in unifocal tumors ≤ 4 cm without extrathyroidal extension or lymph node metastases, lobectomy provides equivalent oncologic outcomes with lower morbidity [20,21,22,23]. A recent study even suggests hemithyroidectomy combined with lateral neck dissection in selected cases with small-volume lateral compartment lymph node metastases [24].

On one hand, as previously discussed, RAI treatment has shown limited utility in low-risk tumors, leading to increasing de-escalation strategies [25]. On the other hand, although total thyroidectomy facilitates thyroglobulin follow-up, most PTC recurrences occur in the cervical region—either in the surgical field or lymph nodes—making high-quality ultrasound performed by experienced radiologists highly effective.

In general, the choice between hemithyroidectomy and total thyroidectomy should primarily be based on the need for RAI administration, which requires complete thyroid removal. Most tumors < 4 cm without additional high-risk features are considered low risk, and RAI is therefore not indicated.

Regarding thyroglobulin monitoring after hemithyroidectomy, it is well known that most recurrences in these patients are locoregional, and expert cervical ultrasound offers high sensitivity for early detection. Consequently, thyroglobulin levels should not be a decisive factor when considering total thyroidectomy in low-risk patients.

**Figure 1:**
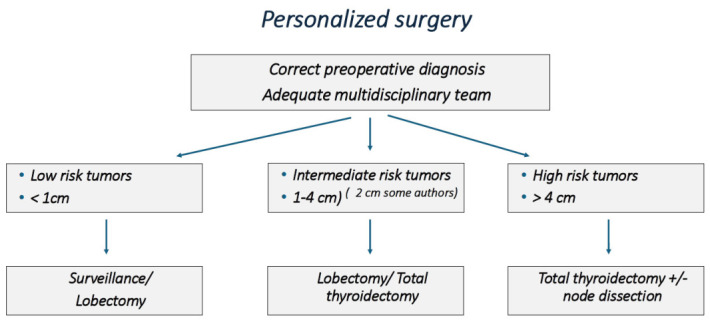
Surgical options in the management of papillary thyroid cancer.

Some recent studies propose lowering the cutoff between hemithyroidectomy and total thyroidectomy to 2 cm without compromising recurrence rates (excluding contralateral lobe recurrence in lobectomy cases) or long-term survival, as tumors larger than 2 cm may show slightly worse outcomes in some hemithyroidectomy series [20]. Although the ATA guidelines initially established 4 cm as a reference cutoff, many authors and societies—and even recent guideline updates—recommend considering a 2 cm threshold to reduce the need for completion thyroidectomy [7] (Figure 1).

Beyond the oncologic rationale for lobectomy, another important factor historically was the high complication rate associated with thyroid surgery performed by non-expert surgeons [26]. Currently, evidence strongly supports that these patients should be treated in high-volume referral centers to minimize surgical complications, ensuring that the choice of surgical extent is based solely on oncologic criteria.

### 4.3. Lymph Node Surgery

Lymph node management depends on the compartment involved (central or lateral) and the presence or absence of preoperative evidence of nodal disease (prophylactic vs. therapeutic).

In the central compartment, prophylactic lymph node dissection remains controversial. Multiple arguments exist for and against its routine use [27], with the prevailing recommendation being to avoid it in low- and intermediate-risk tumors due to its questionable impact on prognosis and survival [25,28,29]. Advocates emphasize more accurate staging and lower postoperative thyroglobulin levels. Occult nodal metastases occur in 40–60% of cases, and reoperation in a previously dissected compartment is technically challenging, which may justify its use to prevent future recurrence.

Over the years, attempts have been made to identify additional factors to guide this decision—such as sentinel lymph node biopsy, BRAF status, or intraoperative biopsy—without achieving clear consensus. Currently, the most widely accepted recommendation is to perform unilateral prophylactic central neck dissection ipsilateral to the tumor in large tumors or those with poor prognostic features, and only when performed by experienced surgeons. When preoperative or intraoperative nodal involvement is evident, bilateral central neck dissection is indicated in all cases.

In the lateral cervical compartment, management is clearer. Prophylactic dissection is not recommended, and surgery should only be performed when nodal involvement is confirmed. In such cases, a functional anatomical neck dissection including at least levels II, III, and IV should be performed, avoiding limited or “atypical” resections.

### 4.4. Endoscopic Surgery in Thyroid Cancer

The conventional cervical approach described by Theodor Kocher remains the gold standard for surgical treatment of PTC due to its low morbidity and mortality when performed in specialized units. However, in recent years, various alternative techniques have emerged aiming to avoid the cervical scar, which is an inevitable outcome in all patients undergoing conventional surgery.

Most of these techniques originated in Asian countries, where extensive experience has been accumulated, particularly in low-risk PTC. These approaches prioritize incision placement outside the cervical region for cosmetic purposes. As a result, several techniques have been developed, including transoral, bilateral axillo-breast, and transaxillary approaches. Although early reports were limited to Asian series with short follow-up, current data show oncologic outcomes comparable to conventional surgery in patients with low- or intermediate-risk tumors, and even in selected cases with limited nodal involvement, provided the procedures are performed in high-volume centers with extensive experience [30,31], but it is true that long-term recurrence data are still lacking.

On the other hand, it is important to emphasize the significant impact on patients’ quality of life. A recent study [32] highlights that despite the excellent prognosis of papillary thyroid cancer, patients may experience a meaningful impairment in quality of life. This impact is mainly driven by psychological factors, particularly fear of recurrence, as well as treatment-related effects. Other studies have similarly reported that anxiety, sleep disturbances, and sequelae of surgery or radioiodine therapy can negatively influence patient-reported outcomes. Altogether, these findings support a more individualized and less aggressive management approach, reinforcing the current trend toward de-escalation in low-risk disease.

## 5. Conclusions and Future Perspectives

For many years, PTC was treated with extensive surgery and routine RAI use, achieving excellent oncologic outcomes but without adequate consideration of overtreatment and long-term sequelae in many patients. It is now well established that a large proportion of tumors—particularly those <2 cm without poor prognostic features—can be managed less aggressively without compromising survival or disease-free outcomes.

Although this is especially relevant in settings where surgery is not performed by expert surgeons, it does not diminish the importance of managing these patients in specialized centers with multidisciplinary teams. Less aggressive surgery or even active surveillance should be carefully evaluated and implemented in such settings.

Deciding the appropriate timing for discharge of patients with papillary thyroid carcinoma (PTC) from specialist follow-up is often challenging and must be individualized. Although most patients with low-risk disease have an excellent long-term prognosis, the risk of late recurrence—albeit low—necessitates a careful balance between prolonged specialist surveillance and transition to primary care. In general, patients classified as low risk, particularly those who achieve an excellent response to initial therapy and remain disease-free after an adequate period of follow-up, may be safely transitioned to monitoring in primary care settings. This shared-care approach allows continued long-term surveillance while reducing the burden on specialist services. However, there is no universally accepted time point for discharge, and decisions should be based on dynamic risk stratification and individual patient factors.

At present, preoperative molecular markers do not yet play a widespread role in patient selection, but they are likely to become an additional valuable tool in the near future.

## Figures and Tables

**Table 1:** Risk of recurrence of papillary thyroid cancer (Adapted from Ringel MD et al. Thyroid. 2025 [7]).

Papillary Thyroid Cancer and Subtypes (WHO 2022 Definitions)	Risk of Recurrence
T3a + microscopic extrathyroidal extension (ETE) T3b T4 Any T with any of… - Poorly differentiated or high grade - Gross incomplete resection (R2) - cN1 ≥ 3 cm - Extranodal extension (ENE) - Distant metastasis (M1)	High (>30%)
T1, T2 or T3a with any of… - Bilateral multifocality > 1 cm - Clinically evident lateral lymph nodes metastasis (cN1b) < 3 cm - Aggressive histology - Vascular invasion - Low-intermediate risk factor x2	Intermediate-High (≥16–30%)
T3a T1 or T2 with any of… - Unilateral multifocality - Microscopic ETE - cN1a or pN1a > 2 mm o > 5 LNs - Negative margins or microscopic + posterior margin (R1)	Low-Intermediate (10–15%)
T1 or T2 (≤4 cm) and… - Unifocal - pN0a or cN0 + pN1a (≤5 LNs, all ≤ 2 mm) - Negative margins or only microscopic + anterior margin (R1)	Low (<10%)

## Data Availability

Not applicable.

## References

[1] Mazzaferri E.L., Young R.L., Oertel J.E., Kemmerer W.T., Page C.P. (1977). Papillary thyroid carcinoma: The impact of therapy in 576 patients. Medicine.

[2] Mazzaferri E.L., Jhiang S.M. (1994). Long-term impact of initial surgical and medical therapy on papillary and follicular thyroid cancer. Am. J. Med..

[3] Davies L., Welch H.G. (2006). Increasing incidence of thyroid cancer in the United States, 1973–2002. JAMA.

[4] Brito J.P., Morris J.C., Montori V.M. (2013). Thyroid cancer: Zealous imaging has increased detection and treatment of low-risk tumors. BMJ.

[5] Vaccarella S., Franceschi S., Bray F., Wild C.P., Plummer M., Dal Maso L. (2016). Worldwide thyroid-cancer epidemic?. N. Engl. J. Med..

[6] Haugen B.R., Alexander E.K., Bible K.C., Doherty G.M., Mandel S.J., Nikiforov Y.E., Pacini F., Randolph G.W., Sawka A.M., Schlumberger M. (2016). 2015 American Thyroid Association management guidelines for adult patients with thyroid nodules and differentiated thyroid cancer. Thyroid.

[7] Ringel M.D., Sosa J.A., Baloch Z.W., Bischoff L., Bloom G., Brent G., Brock P.L., Chou R., Flavell R.R., Goldner W. (2025). 2025 American Thyroid Association management guidelines for adult patients with differentiated thyroid cancer. Thyroid.

[8] Leboulleux S., Bournaud C., Chougnet C.N., Zerdoud S., Al Ghuzlan A., Catargi B., Cao C.D., Kelly A., Barge M., Lacroix L. (2022). Thyroidectomy without radioiodine in patients with low-risk thyroid cancer. N. Engl. J. Med..

[9] Leboulleux S., Bournaud C., Chougnet C.N., Lamartina L., Zerdoud S., Cao C.D., Catargi B., Dygai I., Kelly A., Barge M.L. (2025). Thyroidectomy without radioiodine in patients with low-risk thyroid cancer: 5 years of follow-up of the prospective randomised ESTIMABL2 trial. Lancet Diabetes Endocrinol..

[10] Yang C., Luo D., Xie J., Zou Y., Chen F., Yang W., Zeng L., Liu J. (2025). Is radioiodine necessary for patients with low-risk differentiated thyroid cancer after thyroidectomy: A pooled analysis of ESTIMABL2 and IoN trials. Front. Onco..

[11] Schmid D., Ricci C., Behrens G., Leitzmann M.F. (2015). Adiposity and risk of thyroid cancer: A systematic review and meta-analysis. Obes. Rev..

[12] Agrawal N., Akbani R., Aksoy B.A., Ally A., Arachchi H., Asa S.L., Auman J.T., Balasundaram M., Balu S., Baylin S.B. (2014). Integrated genomic characterization of papillary thyroid carcinoma. Cell.

[13] Liu R., Zhu J., Jiang B., Hu Y., Xue J., Liu W., Yin Y., Wei Y., Sun Z., Li P. (2025). Clinicopathological and molecular characteristics of papillary thyroid carcinoma with BRAF V600E and TERT promoter co-mutations. Pathol. Res. Pract..

[14] Brumfield A., Abou S., Nordgren R., Cohen R., Sarne D., Keutgen X., Applewhite M., Angelos P., Cipriani N.A. (2025). Prevalence and clinical impact of BRAF p.V600E mutation in papillary thyroid carcinoma. Endocr. Pathol..

[15] Baloch Z.W., Asa S.L., Barletta J.A., Ghossein R.A., Juhlin C.C., Jung C.K., Lloyd R.V., Mete O., Kakudo K., Nixon I.J. (2022). Overview of the 2022 WHO classification of thyroid neoplasms. Endocr. Pathol..

[16] Ito Y., Miyauchi A., Inoue H., Fukushima M., Kihara M., Higashiyama T., Tomoda C., Takamura Y., Kobayashi K., Miya A. (2010). An observational trial for papillary thyroid microcarcinoma in Japanese patients. World J. Surg..

[17] Miyauchi A., Ito Y., Oda H. (2018). Insights into the management of papillary microcarcinoma of the thyroid. Thyroid.

[18] Kim M.J., Moon J.H., Lee E.K., Song Y.S., Jung K.Y., Lee J.Y., Park S., Kim T.H., Kim T.Y., Kim W.B. (2024). Active surveillance for low-risk thyroid cancers: A review of current practice guidelines. Endocrinol. Metab..

[19] Miyauchi A., Kudo T., Ito Y., Oda H., Sasai H., Higashiyama T., Fukushima M., Masuoka H., Kihara M., Miya A. (2018). Estimation of the lifetime probability of disease progression of papillary microcarcinoma during active surveillance. Surgery.

[20] Rodriguez Schaap P.M., Botti M., Otten R.H.J., Dreijerink K.M.A., Nieveen van Dijkum E.J.M., Bonjer H.J., Engelsman A.F., Dickhoff C. (2020). Hemithyroidectomy versus total thyroidectomy for well-differentiated T1–2 N0 thyroid cancer: Systematic review and meta-analysis. BJS Open.

[21] Zhang C., Li Y., Li J., Chen X. (2020). Total thyroidectomy versus lobectomy for papillary thyroid cancer: A systematic review and meta-analysis. Medicine.

[22] Wong R.S.H., Sri Ram T.M., Xia Y., Heng E.H., Jayabaskaran J., Lim Y.H., Tan N.C., Foo Y., Chua H., Goh C.H. (2025). Lobectomy versus total thyroidectomy across 2015 ATA low-to-intermediate-risk papillary thyroid carcinoma. Otolaryngol. Head Neck Surg..

[23] Hao Q., Segel J.E., Vanness D.J., Shen C., Hao J., Hollenbeak C.S. (2025). Hemithyroidectomy versus total thyroidectomy for differentiated thyroid cancer: Systematic review and meta-analysis. Gland. Surg..

[24] Wu S., Zhao Y., Li H., Zhang G., Yang H., Fu X., Wang X., Liu J., Chen Y., Zhang L. (2025). Total thyroidectomy versus lobectomy for unilateral papillary thyroid cancer with lateral lymph node metastasis: Systematic review and meta-analysis. Surgery.

[25] Goldfarb M., Ullman N. (2025). Continuing De-escalation Trends: Is Adjuvant Radioactive Iodine Administration Truly Needed for Papillary Thyroid Carcinoma with Limited Cervical Disease?. Ann. Surg. Oncol..

[26] Hsiao V., Light T.J., Adil A.A., Tao M., Chiu A.S., Hitchcock M., Nguyen K., Patel S., Singh A., Brown J. (2022). Complication rates of total thyroidectomy vs hemithyroidectomy for papillary thyroid microcarcinoma: A systematic review and meta-analysis. JAMA Otolaryngol. Head Neck Surg..

[27] Sitges-Serra A., Lorente L., Mateu G., Sancho J.J. (2015). Central neck dissection: A step forward in the treatment of papillary thyroid cancer. Eur. J. Endocrinol..

[28] Papini P., Rossi L., Matrone A., De Renzis A., Morganti R., Valerio L., Borson-Chazot F., Schlumberger M., Barczyński M., Sippel R.S. (2025). Prophylactic central neck dissection in clinically node-negative papillary thyroid carcinoma: 10-year impact on outcomes. Surgery.

[29] Zhao P., Liang L.L., Luo Y.B., Liang Q.K., Xiang B.D., Wang Y., Liu Z., Chen H., Zhang Y., Sun J. (2025). Effectiveness of prophylactic central compartment neck dissection following hemithyroidectomy in papillary thyroid cancer: A meta-analysis. ANZ J. Surg..

[30] Pan J.H., Zhou H., Zhao X.X., Ding H., Wei L., Qin L., Zhang F., Liu M., Wang Q., Chen Y. (2017). Robotic thyroidectomy versus conventional open thyroidectomy for thyroid cancer: Systematic review and meta-analysis. Surg. Endosc..

[31] Nguyen V.C., Song C.M., Ji Y.B., Myung J.K., Park J.S., Tae K., Kim S.Y., Lee H.S., Choi Y., Han W. (2024). Remote-access and minimally invasive video-assisted approaches in lateral neck dissection for papillary thyroid carcinoma: Systematic review and network meta-analysis. Eur. J. Surg. Oncol..

[32] Çıkın C., Bahçecioğlu A.B., Güllü S., Yıldırım S., Kaya M., Demirci T., Öztürk A., Arslan H., Şahin E., Karaca Z. (2025). Assessing quality of life in papillary thyroid cancer: SF-36 versus ThyPRO questionnaire. Sci. Rep..

